# Items

**Published:** 1875-02

**Authors:** 


Alumni Association.—The inaugural meeting of the Association of the Officers and Alumni of the Medical Department of the University of Buffalo, was in every way a success.——State Medical Society.—We are indebted to the *Medical Record* for the full report of the proceedings of the State Society, which we present this month. The selection of Dr. Rochester as President, was an eminently fitting one, and will receive the hearty approval of every physician of Western New York.——Scarlet Fever and the Medical Profession.—The following effusion from the editorial page of the *Commercial Advertiser* of this city needs no comment from us, its utter lack of common sense is self-evident: Scarlet Fever has not raged with as much virulence during the past month as in December and November, if the returns to the Health Physician are any indication. The medical men realized the necessity for action, and the results are already seen. Reading Bancroft's "History of the Native Races" we learn that among some of the tribes on the Northern Pacific coast, the doctor is only called as the last resource. He is given to understand that if he loses the patient he will be killed himself, and if he escapes, then the first medicine-man that the indignant friends of the deceased can lay their hands on is sacrificed. This is somewhat severe; but perhaps if it was tried on now and then among civilized people epidemics would not make such headway.

